# Strengthening anal cancer prevention in Abuja, Nigeria: Identifying barriers and potential strategies to improve training

**DOI:** 10.1371/journal.pgph.0004616

**Published:** 2025-07-02

**Authors:** Connor R. Volpi, John Chama, Megan E. Mansfield, Ruxton Adebiyi, Andrew Mitchell, Jumoke A. Aigoro, Yerima Jibrin Bawa, Kazeem E. Kolawole, Uchenna Ononaku, Paul Jibrin, Oluwole Olaomi, Francis Agbo, Soren M. Bentzen, Stephen E. Goldstone, Patrick Dakum, Joel M. Palefsky, Cheryl Knott, Sylvia Adebajo, Rebecca G. Nowak

**Affiliations:** 1 Department of Health, Behavior and Society, Johns Hopkins Bloomberg School of Public Health, Baltimore, Maryland, United States of America; 2 Institute of Human Virology Nigeria, Abuja, Nigeria; 3 Division of Epidemiology and Public Health, Institute of Human Virology, University of Maryland School of Medicine, Baltimore, Maryland, United States of America; 4 National Hospital, Abuja, Nigeria; 5 National Agency for the Control of AIDS, Abuja, Nigeria; 6 University of Maryland Greenebaum Comprehensive Cancer Center, Baltimore, Maryland, United States of America; 7 Icahn School of Medicine at Mount Sinai, New York, New York, United States of America; 8 University of California, San Francisco, California, United States of America; 9 University of Maryland, College Park, College Park, Maryland, United States of America; Dalhousie University, CANADA

## Abstract

Anal cancer poses a significant risk for sexual minority males (SMM) living with HIV, with a 100-fold higher incidence compared to the general population. Despite success in high-income settings, training on anal cancer prevention in Africa faces challenges due to limited resources and lack of trained practitioners. We evaluated adapting training using an implementation science framework in a Nigerian SMM-friendly clinic. The Consolidated Framework for Implementation Research (CFIR) Card Game assessed barriers to training on anal cancer prevention. Stakeholders ranked the importance of different CFIR constructs. Sessions were conducted separately for internal stakeholders, external stakeholders, and patients. Facilitators identified barriers using culturally adapted text and a hybrid format for consensus discussion. Potential strategies to overcome the barriers were identified with the CFIR-Expert Recommendations for Implementing Change (ERIC) Matching Tool. The CFIR card game was conducted in August 2023 with 20 participants (Internal: 4, External: 8, Patients: 8). Internal stakeholders identified adaptability, cost, and the absence of external change agents. External stakeholders highlighted adaptability, design quality, and financial burden, proposing advocacy and local discussions. Patients expressed concerns about adaptability and external policies affecting trust and acceptance, emphasizing strategic adaptations and local involvement. The CFIR-ERIC Matching Tool recommended identifying champions and altering incentives as strategies. Incorporating champion roles, local adaptations, policy enforcement, and financial support can enhance training on anal cancer prevention in Nigeria. The findings stress the importance of cultural sensitivity and engagement with local stakeholders to support training practitioners in anal cancer prevention.

## Introduction

The risk of anal cancer is 100-fold higher among sexual minority males (SMM) living with HIV compared to the general population [1–5]. In 2022, ANCHOR, a recent randomized controlled trial conducted in the United States, established that treatment of anal high-grade squamous intraepithelial lesions (HSIL), the precursors to anal cancer identified with high resolution anoscopy (HRA), was an effective intervention for anal cancer prevention [6]. However, learning to identify and treat HSIL with HRA requires tailored mentorship and years of training [7]. Furthermore, the number of clinics performing HRA has been limited and arisen independently in settings with available resources [8]. Most anal cancer prevention research, therefore, has originated primarily in high-income countries (HIC) [5].

While the HIV epidemic is concentrated in Africa and there is a rising incidence of cancers caused by human papillomavirus (HPV), including anal cancer, it is prudent that we expand diagnosis and treatment of HSIL to Africa. There is a growing disparity in HPV-associated cancers in Africa as compared to HIC settings [9]. In Nigeria, the national prevalence of HIV is estimated to be 1.4% and SMM account for nearly 20% of new HIV infections nationally [10–12]. Moreover, prevalence of anal high-risk HPV is 70% among Nigerian SMM living with HIV, consistent with other SMM living with HIV outside of Africa [13,14]. Data on the specific prevalence of anal cancer in Africa is limited as it is seldom reported on national cancer registries [15] and screening is unavailable [16].

In Nigeria, there is a robust network of clinics that provide care for persons living with HIV (PLHIV) due to the support of PEPFAR and the Global Fund that collectively fund 94% of Nigeria’s national HIV treatment programs [17]. In Nigeria, we have shown it is feasible and acceptable to integrate cancer prevention in a PEPFAR-supported SMM-friendly HIV clinic [18] despite the high levels of stigma and criminalization [19,20]. However, throughout the implementation of anal cancer screening, we encountered various impediments hindering the attainment of cancer prevention efficacy comparable to that observed in HIC settings. For instance, after conducting over 500 HRAs, the local physician at our Nigerian SMM-friendly clinic only detected an HSIL prevalence of 6% in contrast to the expected prevalence of 30–41% for a clinic learning to detect and treat HSIL [21]. Challenges that arose were in part related to reliance on remote mentoring from colleagues as on-site training was unavailable due to the lack of HRA-practitioners in Africa.

To address our prior gaps, we initiated the Integrated Model for Prevention of Anal Cancer using screen and Treat for HSIL (IMPACT) study to accelerate the learning process on anal cancer prevention for practitioners in Nigeria. One of the objectives of IMPACT is to reintroduce training on HRA in our SMM-friendly clinic and formally evaluate barriers to learning how to detect and treat HSIL using an implementation science framework, the Consolidated Framework for Implementation Research (CFIR) [22]. CFIR offers a practical guide to evaluate challenges utilizing 39 domains that include research constructs such as: intervention source, adaptability, and patient needs and resources [22]. To readily quantify the 39 domains, we used the CFIR Card Game [23]. The CFIR Card Game enables stakeholders in a participatory and engaging format to collaboratively assess implementation challenges that can be readily matched to evidence-based strategies using the CFIR-ERIC Matching Tool v.1. This interactive approach is particularly useful in pre-implementation planning phases, especially in settings where traditional academic frameworks may be inaccessible or difficult to translate into action [23]. This paper reports on the initial barriers to learning and top strategies identified by stakeholders from both internal and external environments, as well as patients who underwent HRA. These insights capture a range of perspectives on how to best train our current and future SMM-friendly clinics on anal cancer prevention in other low-resource settings.

## Materials and methods

### Study setting

The TRUST research clinic partnered with the International Center for Advocacy and Rights to Health (ICARH), an SMM-led community-based organization located in Abuja, Nigeria. By integrating research with an SMM-led community-based organization in 2012, we have been able to establish a cohort of nearly 2,800 SMM, the largest in Africa. Through this partnership, members of the SMM community access friendly HIV prevention and treatment services in a safe and trusted environment. As highlighted in *Science*, the TRUST/ICARH clinic has made seminal contributions to research on the health needs of the SMM community, culminating in more than 50 peer-reviewed publications [24]. The IMPACT study is currently being conducted at TRUST/ICARH.

### Training on HSIL detection and treatment

The IMPACT anoscopist, nurse, and study manager, all intimately involved with HSIL detection and treatment, completed a 9-module online didactic training course (approximately 19 hours) from the International Anal Neoplasia Society (IANS). Next, the research team began experiential learning where they independently initiated anal cancer prevention based on the IANS online training. In the first two weeks of initiation, the IMPACT team consulted with the prior TRUST nurse and surgeon who were involved with HRA in 2016 for initial on-site mentoring [25]. TRUST/ICARH began recruiting eligible SMM for IMPACT in May 2023.

By June 2023, the IMPACT team completed a minimum of 50 evaluations using HRA and were visited by a trained expert from the United States (U.S.) for a week of in-person education and training. Subsequently, the IMPACT team continued detecting HSIL with online consultations from the U.S. expert until they reached 100 HRAs. One hundred HRAs set the cutoff time of when the team would initiate the CFIR card game for evaluation of initial barriers with training as it was determined to be a quality assurance metric for experiential learning by a new anoscopist as per the IANS guidelines [26].

### Card game participants

Purposeful sampling [27] was utilized to engage a range of perspectives both within and beyond the SMM-friendly HIV clinic. The patients, internal, and external stakeholders were separated into three different card game sessions to ensure open and honest discussions, recognizing that perspectives might vary even within an SMM-friendly clinic. Of note, the internal stakeholders included four members of TRUST/ICARH, and external stakeholders included eight persons from various organizations including: the National Hospital, ICARH, National Institute of Cancer Research and Treatment (NICRAT), National Agency for the Control of AIDS (NACA), Federal Capital Territory Agency for the Control of AIDS (FACA), Federal Ministry of Health (FMoH), and a representative from a non-governmental entity (Table 1).

**Table 1:** Participants from each (n = 3) Card Game.

Group	Participant	Affiliation		
**Internal Stakeholders**	Clinic Data Manager	TRUST		
8 August 2023	Clinic Nurse Provider	TRUST		
Virtual	Clinic Director	TRUST		
	Clinic Medical Doctor	TRUST		
**External Stakeholders**	Surgeon	National Hospital		
24 August 2023	Pathologist	National Hospital		
In-person	Program Manager	ICARH		
	Executive Director	National Institute of Cancer Research and Treatment (NICRAT)		
	Senior Program Manager	National Agency for the Control of AIDS (NACA)		
	Program Officer	FCT Agency for the Control of AIDS (FACA)		
	Program Officer	Federal Ministry of Health (FMoH)		
	Director	Representative of Private Sector		
**Screened Patients**		**CFIR Criteria**	**Pain Score**	**ART Adherence**
22 August 2023	Patient 1	Good ART	4	≥95% of pills
In-person	Patient 2	Good ART	1	≥95% of pills
	Patient 3	Moderate ART	5	50-95% of pills
	Patient 4	Moderate ART	2	50-95% of pills
	Patient 5	High pain	8	50-95% of pills
	Patient 6	High pain	8	≥95% of pills
	Patient 7	Low pain	1	50-95% of pills
	Patient 8	Low pain	1	≥95% of pills

To capture the perspectives from patients with varying health behaviors and different experiences with HRA, we used antiretroviral adherence and HRA-related pain variables. We invited eight patients who self-reported high (≥95% of pills) or moderate (50–95% of pills) adherence to antiretroviral therapy at enrollment. We further stratified those patients by whether they reported high or low levels of pain in their post HRA satisfaction survey.

### Data collection

The CFIR Card Game [23] was played by each stakeholder group to identify the perceived barriers to HRA training. Of note, certain cards were not used during the session when not applicable for that specific group (e.g., participants who participated in HRA were not asked about training logistics). The text used during the game was culturally adapted to address linguistic differences specific to the setting. The Card Game was performed solely in-person for patients to garner privacy whereas it was hybrid (in-person or online) for the internal and external stakeholders. Each game was facilitated by two members of the IMPACT team with one leading and the other observing and documenting the proceedings.

During the hybrid sessions, IMPACT principal investigators played a supportive role by reassuring each group that identifying challenges was a normal part of the process and doing so would enable the development of effective strategies to overcome them. During each round, card game players took turns in selecting a card from the central deck, reading its content aloud to the group. The corresponding text was projected, ensuring that players could both hear and read the cards for enhanced comprehension. Subsequently, the group deliberated on whether the described statement posed a challenge to learning HRA.

**Fig 1:**
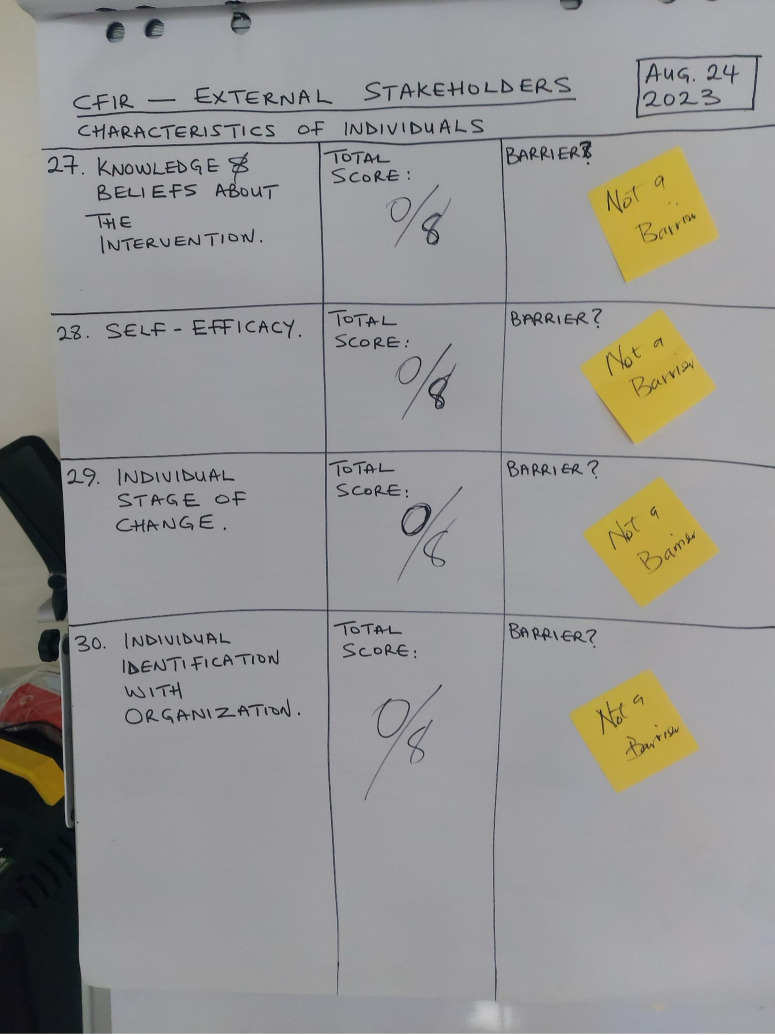
Barriers Identification Process.

While consensus was the preferred method for determining if a card was a barrier, a vote through a show of hands was used if consensus could not be reached within a few minutes. This approach balanced the need for efficiency with inclusivity, ensuring that all participants, including patients, had an opportunity to contribute meaningfully without the process becoming overly time-consuming. The facilitator encouraged players to share their reasons and thought processes with others in the group, fostering a deeper understanding of the perceived challenges. The facilitator quantified barriers (S1 Table) through written documentation and player votes, which were subsequently confirmed using sticky post it notes to summarize the groups consensus (Fig 1). As per the card game [23], if at least half of the players (≥50%) vocalized concerns over that characteristic, then it was deemed a barrier. Barriers were collated within the specific groups (i.e., patients, internal, and external stakeholders) and were included in that group’s respective analysis.

To capture the dynamics of the game, the process was documented in real-time using the Observation Template developed by CFIR [23] (S2 Table; https://cfirguide.org/tools/tools-and-templates/). This sheet recorded player responses and any noteworthy observations, such as silences or disagreements (S3 Table). Additionally, photographs were taken of the poster boards, serving as a visual record of each team’s responses and the evolving landscape of identified challenges. This comprehensive methodology ensured a systematic and interactive approach to exploring barriers related to HRA training. Subsequent to the player group discussions, the research team inputted the barriers from each respective group into the CFIR-ERIC Matching Tool v.1 [28], resulting in a ranking of implementation strategies endorsed by experts that could simultaneously overcome the inputted barriers. The Standards for Quality Improvement Reporting Excellence (SQUIRE) [29] were utilized to ensure comprehensive and transparent reporting throughout the study period.

### Ethical considerations and inclusivity in global research

This study was reviewed and approved by the Federal Capital Territory Health Research Ethics Committee [FHREC/2022/01/143/26-07-22], and the National Health Research Ethics Committee of Nigeria [NHREC/01/01/2007-03/08/2022], the Data & Safety Monitoring/Quality Assurance Committee and the Clinical Research Committee at the University of Maryland Greenebaum Comprehensive Cancer Center [2267GCCC], and the University of Maryland Baltimore IRB [HP-00101383]. All participants gave their written informed consent to participate in this research project. Additional information regarding the ethical, cultural, and scientific considerations specific to inclusivity in global research is included in the Supporting Information (S1 Checklist).

## Results

### Internal stakeholders

The internal stakeholders identified ten barriers to learning HRA within 5 CFIR domains. Barriers related to intervention characteristics (HRA training) included adaptability, trialability, and cost. Barriers related to the outer setting (external environment) included peer pressure, external policy and incentives. Barriers related to the inner setting (internal environment) included organization incentives & rewards and available resources. Barriers related to characteristics of the individuals were individual stage of change and barriers related to the process included external change agents and key stakeholders (Table 2, S1 Table). In reference to adaptability as a barrier, players voiced concern about practicality and the lack of expertise and hands-on training available locally. When they expanded on cost and available resources as a barrier, players described financial constraints such as sustainable affordability, inadequate infrastructure, and the need for local modifications. In reference to trialability, internal stakeholders voiced concerns that the significant investment in online training and mentorship programs limited testability on a small scale. They highlighted that once these programs were implemented, it would be hard to retract or modify training without significant loss in effort and resources.

**Table 2:** Identified barriers separated by groups.

CFIR Barrier	Internal Stakeholders	External Stakeholders	Screened Patients
**Intervention Characteristics**			
Adaptability	x	x	x
Trialability	x		
Design quality and packaging		x	
Cost	x	x	
**Outer Setting**			
Patient needs and resources		x	
Cosmopolitanism		x	
Peer pressure	x	x	x
External policy & incentives	x	x	x
**Inner Setting**			
Organizational incentives & rewards	x		
Available resources	x		
**Characteristics of Individuals**			
Individual stage of change	x		
**Process**			
Champions	x		
External change agents	x		
Key stakeholders	x		

Other concerns from the team involved with training included not having external change agents in the external environment who could locally support HRA training, regardless of the stigma and discrimination faced by SMM in Nigeria. Team members raised apprehensions about the feasibility of expanding training outside of the TRUST clinic, especially if the benefit were limited to a subset of the general population. Internal stakeholders also noted that the training guidelines, largely developed in the U.S., lacked relevance for the Nigerian context and appeared externally imposed, raising apprehension about replicating historical patterns of top-down, non-localized healthcare interventions and potentially hindering adaptability.

Internal stakeholders expressed concerns that the skills they were acquiring were not widely valued beyond PEPFAR-funded sites in Nigeria. However, this challenge could be addressed by fostering collaborations with external partners who, with their understanding of local culture and infrastructure, could help refine messaging to emphasize the importance of screening and align it with national health priorities. Although some expressed confidence with the training, others highlighted practical challenges and the need for sustained contact with experienced mentors, financial support, and local advocacy for the acceptance of trained practitioners into the existing healthcare framework. Based on these barriers, the CFIR-ERIC Matching Tool v.1 recommended several implementation strategies with the top three including: alter incentives and allowance structures, identify and prepare champions, and access new funding (Table 3).

**Table 3:** Top three recommended strategies from the CFIR-ERIC Matching Tool.

CFIR-ERIC Strategy	Internal Stakeholders	External Stakeholders	Screened Patients
Alter incentive/allowance structures	1	--	1
Identify and prepare champions	2	--	2
Access new funding	3	--	--
Promote adaptability	--	1	--
Conduct local consensus discussions	--	2	3
Conduct local needs assessment	--	3	--

### External stakeholders

The external stakeholders pinpointed seven barriers to training on HRA within two CFIR domains. Barriers related to intervention characteristics included adaptability, design quality and packaging, and cost. Barriers related to the outer setting included patient needs & resources, cosmopolitanism, peer pressure, and external policy and incentives (Table 2, S1 Table).

With respect to adaptability and design quality and packaging, external stakeholders expressed skepticism about the U.S.-centric origins of the training guidelines, particularly the ANCHOR protocol, questioning their applicability to Nigeria’s healthcare system and cultural context. This skepticism was rooted in broader concern about colonial legacies in medical training and underscored the need for implementation strategies that are locally adapted and community-owned. Participants noted that work specifically done in Nigeria, such as the IMPACT study, could overcome these barriers to learning HRA. It was noted that the number of steps needed to achieve proficiency, particularly steps related to mentorship, was a potential obstacle given no local mentors. However, external stakeholders did not rank trialability as a barrier because the training brought innovation to Nigeria.

External stakeholders also noted that the associated costs, encompassing various aspects of patients’ needs and resources (e.g., infrastructure, training, and instrumentation) could pose challenges for widespread training. Cosmopolitanism, representing the extent to which the clinic and its staff are connected to broader networks and communities outside their local setting, was initially discussed as a potential barrier. However, the IMPACT study’s established connections with international experts who developed the training guidelines mitigated concerns about isolation from the wider HRA community. During the discussion, external stakeholders noted that there was no peer pressure from the Nigerian healthcare settings to learn anal cancer prevention. Without that external peer pressure, seamless adoption of training on HRA would be hampered. The financial burden associated with training, travel, and equipment was further discussed as exacerbating these challenges and appeared specific to external policy and incentives.

External stakeholders noted that the Western-centric nature of the guidelines may limit their effectiveness in addressing the multifaceted needs of patients in non-Western settings, potentially undermining their engagement with HRA and ultimately limiting the number of patients needed for the training practitioner. This barrier could exacerbate peer pressure from healthcare settings in Nigeria to advocate for change and adopt the training guidelines, thus, further impeding expansion of trained practitioners. The external stakeholders noted that overcoming these barriers may necessitate not only financial support but also strategic adaptations, local involvement, and policy enforcement to ensure broader acceptance for this innovative training. Derived from these identified barriers, the top three strategies were to advocate for adaptability, initiate local consensus discussions, and conduct local needs assessments as recommended by the CFIR-ERIC Matching Tool v.1 (Table 3).

### Screened patients

Finally, patients who have undergone HRA identified three pivotal concepts representing potential hurdles in the training and implementation of HRA that fell within two CFIR domains. The obstacles were 1) adaptability [within the intervention characteristics domain] and 2) peer pressure and 3) external policy and incentives [both within the outer setting domain] (Table 2, S1 Table).

The sentiment that the procedure is perceived as unique and not developed in an African setting raised cultural and contextual considerations for patients brought up concerns related to the adaptability of training on HRA in this setting. Apprehension was raised about convincing Nigerians of a practitioner trained in anal cancer prevention that may not align with established norms or practices. Furthermore, the notion that other places may not be deemed safe for adapting the training (e.g., anti-SMM) was emphasized as being crucial for its acceptance, ultimately hindering Nigeria from creating a beneficial peer pressure for expansion of training.

Participants also noted that external policies and pervasive stigma, particularly fear of discrimination when seeking care as SMM, could deter engagement and contribute to patients actively discouraging peers from participating in HRA, ultimately hindering the training process, which relies heavily on consistent patient flow. Participants highlighted the potential hurdle of ensuring proper training for staff, indicating that the success of training hinges on the availability of skilled personnel. The resistance to adopt a new health behavior without external pressure from health authorities in Nigeria suggests a prevailing reluctance to engage with a trained practitioner unless compelling reasons are presented. Lastly, the concern about trust in the clinic staff, even when trained, highlights the nuanced dynamics of acceptance and underscores the need for broadly building confidence in the doctor-patient relationship. In response to these identified barriers, the CFIR-ERIC Matching Tool v.1 put forth a range of recommended strategies, with the foremost three being to alter incentives and allowance structures, identify and prepare champions, and initiate local consensus discussions (Table 3).

## Discussion

Our study utilized the CFIR card game to identify barriers to HRA training [23]. We conducted three card games among clinic personnel involved with HRA, patients who underwent HSIL detection, and physicians and leaders from national hospitals and government agencies to understand how to best incorporate training within clinics and Nigeria. These efforts address the urgent need for effective and sustainable training on anal cancer prevention, particularly for SMM living with HIV, who, despite limited geographic specific data [15,16], are known to face a disproportionately high risk [1–5]. Although ANCHOR demonstrated the efficacy of treating HSIL to prevent anal cancer [6], there exists a critical gap in the literature on how to expand and teach the efficacious cancer prevention procedure to Africa. Notably, a 2021 meta-analysis revealed a dearth of research on anal cancer in non-Western settings, underscoring the necessity for targeted training in these regions [5].

The IMPACT study in Nigeria represents an effort to bridge this gap by assessing how to train on HRA in a non-western setting with limited resources and high anti-SMM sentiment. As the study unfolds, its primary objective is to equip the clinic with sustainable cancer prevention which can ultimately be used to guide national cancer prevention efforts. To achieve this, the current research employed the CFIR Card Game, involving key stakeholders, both internal and external, to identify barriers related to the training procedures in the early phases of learning HRA.

The identified barriers encompass a range of challenges, from skepticism about the Western origins and limited contextual relevance of the training guidelines, to practical concerns about adaptability and local expertise. Findings specific to skepticism relate to the perception some Nigerians may have on colonial medical practices and historic inequities that have taken place on the African continent [30,31]. Furthermore, financial constraints, infrastructural inadequacies, and the need for local modifications emerged as formidable hurdles to becoming a trained practitioner, compounded by limited access to online training and mentorship programs. To overcome these challenges, early-stage donor support could be leveraged to build local capacity, while advocating for phased integration into national training systems. Long-term sustainability will require not only financial investment but also policy alignment and political will, particularly in settings where stigma and legal constraints may hinder government engagement in programs serving populations at-risk. Given the global trend of donor funding and shifting priorities, there is an urgent need to diversify funding strategies. As highlighted in recent analyses [32], increasing domestic resource mobilization, such as through health taxes or insurance schemes, and leveraging public-private partnerships can reduce dependency on external aid while promoting long-term sustainability.

Interestingly, the internal stakeholders identified more training barriers overall and across more domains than the other two groups. This is likely due to the intimate involvement of internal stakeholders with the components of the guidelines and having learned from the IMPACT study that their objective is to find strategies to optimize the training. The external team added valuable insights on the perspective of the Nigerian context and highlighted that the innovative training was trialable, potentially leading to greater acceptance in the broader healthcare context.

One recurrent theme was the skepticism surrounding training guidelines developed outside Nigeria, highlighting the necessity for contextual relevance and local contributions to enhance the guideline applicability. Concerns about ANCHOR’s [33] U.S.-centric origins and an existing infrastructure of trained practitioners further emphasized the need for comprehensive strategies that account for building up a team of trained personnel. Additionally, the complexity of training in HRA is acknowledged as both an obstacle and an advantage, demanding careful consideration and strategic planning. This call for consideration was recently identified by Blair et al. who highlighted the urgent need for data specific to implementation strategies in non-Western settings to inform adoption of anal cancer prevention in historically underserved settings [16]. The intersection of novel implementation and cultural involvement in training guidelines is in alignment with the literature where cultural adaptation has obtained a high level of ecological validity and external validity for an intervention in implementation science [34,35]. This increased validity could help ensure that the introduction of training on anal cancer prevention succeeds at the TRUST/ICARH clinic and elsewhere in Nigeria.

Patients’ perspectives shed light on cultural considerations and emphasized the uniqueness of the procedure yet the challenge of convincing Nigerians to adopt a prevention method perceived as foreign. With patients disengaged and spreading messages with their friends about their personal hesitancy, this will hinder any training of a practitioner who relies on a high patient flow to learn. The resistance to change without health recommendations from HIV-related national agencies underscores the need for compelling reasons and effective advocacy to foster confidence among SMM to engage in cancer prevention. Trust in staff, even when trained, emerged as a crucial factor, which further emphasizes the nuanced dynamics of acceptance and the imperative to build confidence in the local safe space environment [36]. This finding aligns with calls for the increased training of healthcare workers to help care for SMM [37,38], which is particularly needed in Africa [39] where increased levels of stigma may impede an individual’s ability to engage in the healthcare system [40,41].

Interestingly, the top two recommended strategies from the CFIR-ERIC Matching Tool v.1 for both the internal stakeholders and patients who engaged in HRA were to alter incentives and allowance structures and identify and prepare champions. Altering incentives and allowance structures can play a pivotal role in preparing champions by providing tangible rewards or recognition for their efforts in promoting and implementing initiatives such as HRA training. The role of a champion is akin to an advocate, whose role in achieving other health outcomes is well-documented throughout Africa and has been historically utilized to bring awareness to a given public health issue [42–44]. The identification and usage of a champion could be a novel way to ensure the learning process in this context [45]. This could be particularly helpful for SMM in Nigeria where research has shown the benefit of the utilization of key influencers in health outcomes [46,47]. Further, the identification of a champion who can advocate for the trained HRA practitioners and bring awareness to the fact that the clinic is a safe-space for SMM is in alignment with the needs of the community [48,49].

The main limitations of this study revolve around the application of the CFIR and the CFIR ERIC Matching Tool v.1. Firstly, the evaluation of participants’ full understanding of a given barrier and ability to vocalize additional barriers was limited. However, participants were given additional context to each of the barriers to improve comprehension and were translated to the local language of Hausa when needed. Secondly, the developers of the game highlight that it deviates from a strengths-based recovery-oriented approach by framing the game around challenges rather than facilitators. Additional in-depth qualitative work is forthcoming to further examine the facilitators. Power dynamics emerged during the card game sessions where the facilitator and individuals who expressed their lived experiences first potentially biased the groups’ consensus-building. However, the facilitator for each game tried to be mindful of a person’s desire to speak first to set their opinion at the top of the discussion. We also note that the data reflect only whether a given barrier reached majority agreement within each stakeholder group, in line with the structured approach of the CFIR card game. As such, we did not capture the full distribution of individual responses, including minority or dissenting views. This method may have masked within-group variability and limited our ability to assess the strength of consensus or disagreement on specific issues. Lastly, this study used v1 of CFIR-ERIC Matching Tool which may need further testing and consideration as the tool evolves. Furthermore, its rapid output of respective strategies allows for real time modifications to training to promote continued learning of HRA and anal cancer prevention.

## Conclusion

The findings from this study highlight the complexities of training practitioners in anal cancer prevention in Nigeria, while also offering actionable insights to address these challenges. Moving forward, we propose a framework to guide the development and implementation of training programs tailored to the local context. Central to this effort is a culturally adapted training curriculum aimed at enhancing practitioner expertise in HRA and associated prevention methods. We propose the following components:

1. **Modular Training Materials**: Incorporating local case studies, visual aids, and role-playing exercises that address identified barriers, such as trust and adaptability to local context.
2. **Champion-Driven Advocacy**: Identifying and empowering local healthcare leaders to promote training uptake and foster trust within the community.
3. **Incentive Structures**: Offering financial and professional development incentives to practitioners to encourage participation and retention.
4. **Policy Integration**: Collaborating with policymakers to build consensus to champion the innovative training in support of anal cancer prevention efforts.
5. **Stakeholder Engagement Platforms**: Establishing regular feedback loops with patients, practitioners, and external stakeholders to refine training based on real-world experiences.

Regarding the CFIR card game, this tool facilitated structured discussions by presenting stakeholders with key constructs (e.g., adaptability, cost) on individual cards. Participants ranked these constructs based on perceived importance to anal cancer prevention training. Unlike a traditional questionnaire or lecture, the card game fostered active collaboration, consensus-building, and prioritization of barriers in a visually engaging and participatory manner. By using the card game, we aimed to produce a replicable, context-specific model for tailoring anal cancer prevention training in resource-limited settings. This approach broadly covered inclusivity, cultural relevance, and stakeholder-driven solutions, ensuring the sustainability and scalability of HRA training efforts in Nigeria and similar lower resource contexts.

## Supporting information

S1 Table Definitions for 39 CFIR Barriers.
(DOCX)

S2 Table The CFIR Game Observation Template.
(DOCX)

S3 Table Raw Data. (DOCX)

S1 Checklist Inclusivity in Global Research Questionnaire. (DOCX)
