# Supplementary material for: Strengthening anal cancer prevention in Abuja, Nigeria: Identifying barriers and potential strategies to improve training
Source: PLOS Glob Public Health. 2025 Jul 2;5(7):e0004616. doi: 10.1371/journal.pgph.0004616 (PMC12221040; doi:10.1371/journal.pgph.0004616)
Supplement: S1 Table — Definitions for 39 CFIR Barriers. (DOCX) [file pgph.0004616.s001.docx]

**S1 Table**. Definitions for 39 CFIR Barriers

| **No.** | **Barrier** | **Definition** |
| --- | --- | --- |
|  | **Intervention Characteristics** | |
| 1 | Intervention Source | Team members do not like the IANS guidelines because of who developed them or where they were developed. |
| 2 | Evidence Strength & Quality | Team members do not like the IANS guidelines because they question the research data that says they are helpful for patients. |
| 3 | Relative advantage | Team members do not see why the IANS guidelines are better than what they are already doing or other guidelines they could try. |
| 4 | Adaptability | Team members do not think the IANS guidelines can be adapted to local needs. |
| 5 | Trialability | Team members do not think they can pilot the IANS guidelines or revert to the status quo. |
| 6 | Complexity | Team members believe the IANS guidelines are complex because they involve lots of steps, take a long time, or are disruptive. |
| 7 | Design Quality and Packaging | Team members believe the IANS guidelines are not very good because of the way they have been packaged and presented. |
| 8 | Cost | Team members think the IANS guidelines would be too expensive to implement. |
|  | **Outer Setting** | |
| 9 | Patient Needs & Resources | The IANS guidelines do not account for what patients in Nigeria need, or do not address how to meet those needs. |
| 10 | Cosmopolitanism | The IANS organization is not well connected to partners in Nigeria. |
| 11 | Peer Pressure | There is no pressure to implement the IANS guidelines because other clinics in Nigeria are not using them. |
| 12 | External Policy & Incentives | There are no clinics or outside rules that say the IANS guidelines must be adopted. Or the rules in place get in the way of adopting the guidelines. |
|  | **Inner Setting** | |
| 13 | Structural Characteristics | The TRUST clinic size, history, or leadership/staffing structure get in the way of adopting the IANS guidelines. |
| 14 | Networks & Communications | It is hard to implement the IANS guidelines because of poor communication in the TRUST clinic. |
| 15 | Culture | The culture of the TRUST clinic (the ways things are done, the values, and the way of thinking) makes implementing the IANS guidelines more difficult. |
| 16 | Implementation Climate | It is hard to change things in the TRUST clinic because people are either not willing to make changes or they do not see the benefit of trying something new. |
| 17 | Tension for Change | Team members do not think the IANS guidelines are needed. |
| 18 | Compatibility | There is not a good fit between the IANS guidelines and the existing workflows, systems, norms and values of the TRUST clinic. |
| 19 | Relative Priority | Team members feel that implementing the IANS guidelines is not given much importance because other activities have a higher priority. |
| 20 | Organizational Incentives & Rewards | There is no incentive or reward for adopting the IANS guidelines, such as salary raises or respect from peers or leadership. |
| 21 | Goals and Feedback | Goals are not clearly shared in the TRUST clinic and team members do not get feedback on progress toward any goals. |
| 22 | Learning Climate | Leaders in the TRUST clinic (executives, middle management, supervisors, team leaders) do not show their weaknesses or ask for help from team members. Team members do not feel valued or supported to try new things, and do not feel they have enough time and space to think about and evaluate their practice. |
| 23 | Readiness for Implementation | It is not clear that the TRUST clinic is ready and committed to implement the IANS guidelines. |
| 24 | Leadership Engagement | Key leaders or managers in the TRUST clinic are not involved in, committed to, or held responsible for the implementation of the IANS guidelines. |
| 25 | Available Resources | There are not enough resources (money, training, education, space, time) to support the IANS guidelines. |
| 26 | Access to knowledge and information | Team members do not have enough access to knowledge and information to help them with putting the guidelines into practice. |
|  | **Characteristics of Individuals** | |
| 27 | Knowledge & Beliefs about the Intervention | Team members do not like the IANS guidelines, do not understand them, and do not put a lot of importance on implementing them. |
| 28 | Self-efficacy | Team members are not confident they can do what is needed to reach implementation goals. |
| 29 | Individual Stage of Change | Team members do not have the skills or are not that excited about continuing to use the IANS guidelines. |
| 30 | Individual Identification with Organization | Team members are not satisfied and are not really committed to the TRUST clinic. |
|  | **Process** | |
| 31 | Planning | A plan for implementing the IANS guidelines, including all the steps needed, has not been made or is poorly outlined. |
| 32 | Opinion Leaders | Gatekeepers or opinion leaders who can influence other peoples’ attitudes or beliefs toward implementing the IANS guidelines are not involved or supportive. |
| 33 | Formally appointed internal implementation leaders | There is no one person(s) responsible for making sure the IANS guidelines are being implemented. |
| 34 | Champions | Individuals who act as champions for the IANS guidelines by supporting, marketing, driving, or overcoming any indifference or resistance from key team members, are not involved or supportive. |
| 35 | External Change Agents | Individuals from an outside organization who help with decision-making and moving things forward, are not involved or supportive. |
| 36 | Key Stakeholders | Ways of attracting and involving key stakeholders in implementing the IANS guidelines (e.g. posters, pamphlets, information sessions, training, role modelling) have not been developed or do not work. |
| 37 | Patients/Customers | Ways of attracting and involving clients in implementing the IANS guidelines (e.g. posters, pamphlets, information sessions, training, role modelling) have not been developed or do not work. |
| 38 | Executing | The IANS guidelines have not been implemented as planned. |
| 39 | Reflecting & Evaluating | There is little or no feedback about how implementation of the IANS guidelines is going, and no regular meetings to talk about progress or peoples’ experiences are being held. |
