# Supplementary material for: Strengthening anal cancer prevention in Abuja, Nigeria: Identifying barriers and potential strategies to improve training
Source: PLOS Glob Public Health. 2025 Jul 2;5(7):e0004616. doi: 10.1371/journal.pgph.0004616 (PMC12221040; doi:10.1371/journal.pgph.0004616)
Supplement: S2 Table — The CFIR Game Observation Template. (DOCX) [file pgph.0004616.s002.docx]

**S2 Table**: The CFIR Game Observation Template

| **No.** | **Barrier** | **Operationalization** | **Comments** |
| --- | --- | --- | --- |
|  | **Intervention Characteristics** | | |
| 1 | Intervention Source |  |  |
| 2 | Evidence Strength & Quality |  |  |
| 3 | Relative advantage |  |  |
| 4 | Adaptability |  |  |
| 5 | Trialability |  |  |
| 6 | Complexity |  |  |
| 7 | Design Quality and Packaging |  |  |
| 8 | Cost |  |  |
|  | **Outer Setting** | | |
| 9 | Patient Needs & Resources |  |  |
| 10 | Cosmopolitanism |  |  |
| 11 | Peer Pressure |  |  |
| 12 | External Policy & Incentives |  |  |
|  | **Inner Setting** | | |
| 13 | Structural Characteristics |  |  |
| 14 | Networks & Communications |  |  |
| 15 | Culture |  |  |
| 16 | Implementation Climate |  |  |
| 17 | Tension for Change |  |  |
| 18 | Compatibility |  |  |
| 19 | Relative Priority |  |  |
| 20 | Organizational Incentives & Rewards |  |  |
| 21 | Goals and Feedback |  |  |
| 22 | Learning Climate |  |  |
| 23 | Readiness for Implementation |  |  |
| 24 | Leadership Engagement |  |  |
| 25 | Available Resources |  |  |
| 26 | Access to knowledge and information |  |  |
|  | **Characteristics of Individuals** | | |
| 27 | Knowledge & Beliefs about the Intervention |  |  |
| 28 | Self-efficacy |  |  |
| 29 | Individual Stage of Change |  |  |
| 30 | Individual Identification with Organization |  |  |
|  | **Process** | | |
| 31 | Planning |  |  |
| 32 | Opinion Leaders |  |  |
| 33 | Formally appointed internal implementation leaders |  |  |
| 34 | Champions |  |  |
| 35 | External Change Agents |  |  |
| 36 | Key Stakeholders |  |  |
| 37 | Patients/Customers |  |  |
| 38 | Executing |  |  |
| 39 | Reflecting & Evaluating |  |  |
