# Supplementary material for: Strengthening anal cancer prevention in Abuja, Nigeria: Identifying barriers and potential strategies to improve training
Source: PLOS Glob Public Health. 2025 Jul 2;5(7):e0004616. doi: 10.1371/journal.pgph.0004616 (PMC12221040; doi:10.1371/journal.pgph.0004616)
Supplement: S3 Table — (DOCX) [file pgph.0004616.s003.docx]

**S3 Table. Raw Data**

**IMPACT CFIR Card Game for Internal Stakeholders**

| Aug.8, 2023.  Time started: 11:00 AM (WAT)  Introduction, presentation and consenting.  Time started CFIR game: 11:43 AM (WAT)  Time ended CFIR game: 2:04 PM (WAT)  Indicates an identified barrier | | | | |
| --- | --- | --- | --- | --- |
| INNOVATION CHARACTERISTICS | |  | Participants (operationalization) | Participants (data) |
| 1 | Innovation Source | Team members don’t like the IANS guidelines because of who developed them or where they were developed. | This is a barrier: 0  Not a barrier: 4  Comment: unanimous | 4/4 |
| 2 | Evidence Strength & Quality | Team members don’t like the IANS guidelines because they question the research data that says they are helpful for patients. | This is a barrier: 0  Not a barrier: 4  Comment: unanimous | 4/4 |
| 3 | Relative advantage | Team members do not see why the IANS guidelines is better than what they are already doing or other guidelines they could try. | This is a barrier: 0  Not a barrier: 4  Comment: unanimous | 4/4 |
| 4 | Adaptability | Team members don’t think the IANS guidelines can be adapted to local needs. | This is a barrier: 4  Not a barrier: 0  Comment: it can be adopted with some modifications to fit to local context. If taking training- need online course. only can do the training for that period online. Way packaged- cannot share with colleagues. To be an expert, need a professional to watch that person to do. can be a challenge for preceptorship. for mentorship need local. cost of barrier to get international experts. standard equipment cannot be adopted locally. don't have resuscitation space. no oncology. could be adopted if modified to fit into local needs. it is a barrier. but can be adopted. | 4/4 |
| 5 | Trialability | Team members don’t think they can pilot the IANS guidelines or revert to the status quo. | This is a barrier: 4  Not a barrier: 0  Comment: Find that using guidelines has trialability. Works in the clinic. But this is a research program. Believe can be tried. If adopt to local context then can pilot. Could be tried at heartland alliance. Take care of local factors. Could be done at other OSSs. could be trialable in other OSSs. if we were to take the guidelines as a complete package- big barrier. success in TRUST b/c of resource support. make trust be the center for training. | 4/4 |
| 6 | Complexity | Team members believe the IANS guidelines are complex because they involve lots of steps, take a long time, and/ or are disruptive. | This is a barrier: 0  Not a barrier: 4  Comment: unanimous | 4/4 |
| 7 | Design Quality and Packaging | Team members believe the IANS guidelines aren’t very good because of the way they have been packaged and presented. | This is a barrier: 0  Not a barrier: 4  Comment: unanimous | 4/4 |
| 8 | Cost | Team members think the IANS guidelines would be too expensive to implement. | This is a barrier: 4  Not a barrier: 0  Comment: would need to be funded by government. Need resource of money to make process cheaper. If do colonoscopy. N200,000. HRA could be N100,000. Higher income SGM could possibly pay. But lower SES- need something to stimulate them to pay. If positive on cytology, then would go beyond way beyond his pocket. need to be subsidized to be successful business model. definitely a barrier. | 4/4 |
| OUTER SETTING | |  |  |  |
| 9 | Patient Needs & Resources | The IANS guidelines don’t account for what patients in Nigeria need, or don’t address how to meet those needs. | This is a barrier: 0  Not a barrier: 4  Comment: unanimous | 4/4 |
| 10 | Cosmopolitanism | The IANS organization is not well connected to partners in Nigeria. | This is a barrier: 0  Not a barrier: 4  Comment: unanimous | 4/4 |
| 11 | Peer Pressure | There is no pressure to implement the IANS guidelines because other clinics in Nigeria are not using them. | This is a barrier: 4  Not a barrier: 0  Comment: [NAME] goes first. Not connected with outside partners. Barrier. Would be better if connected to heartland alliance. Other CBOs. Would help with sensitization. Talk about eligibility for screening to increase referrals. [NAME] says barrier. Connectivity with outside partners (key pops) we have some connections, but limited. law has restricted us from being connected to partners. will limit our ability to expand enhanced training. [NAME] agrees that not well connected. | 4/4 |
| 12 | External Policy & Incentives | There are no clinic or outside rules that say the IANS guidelines must be adopted. Or the rules in place get in the way of adopting the guidelines. | This is a barrier: 4  Not a barrier: 0  Comment: barrier, barrier, barrier, if part of policy then can easily adopt it. if policy makers buy into it then can implement. | 4/4 |
| INNER SETTING | |  |  |  |
| 13 | Structural Characteristics | The TRUST clinic size, history, or leadership/staffing structure get in the way of adopting the IANS guidelines. | This is a barrier: 0  Not a barrier: 4  Comment: unanimous | 4/4 |
| 14 | Networks & Communications | It's hard to implement the IANS guidelines because of poor communication in the TRUST clinic. | This is a barrier: 0  Not a barrier: 4  Comment: unanimous | 4/4 |
| 15 | Culture | The culture of the TRUST clinic (the ways things are done, the values, and the way of thinking) makes implementing the IANS guidelines more difficult. | This is a barrier: 0  Not a barrier: 4  Comment: unanimous | 4/4 |
| 16 | Implementation Climate | It's hard to change things in the TRUST clinic because people are either not willing to make changes or they do not see the benefit of trying something new. | This is a barrier: 0  Not a barrier: 4  Comment: unanimous | 4/4 |
| 17 | Tension for Change | Team members do not think the IANS guidelines are needed. | This is a barrier: 0  Not a barrier: 4  Comment: unanimous | 4/4 |
| 18 | Compatibility | There isn’t a good fit between the IANS guidelines and the existing workflows, systems, norms and values of the TRUST clinic. | This is a barrier: 0  Not a barrier: 4  Comment: unanimous | 4/4 |
| 19 | Relative Priority | Team members feel that implementing the IANS guidelines isn’t given much importance because other activities have a higher priority. | This is a barrier: 0  Not a barrier: 4  Comment: unanimous | 4/4 |
| 20 | Organizational Incentives & Rewards | There is no incentive or reward if adopt the IANS guidelines, such as salary raises or respect from peers or leadership. | This is a barrier: 4  Not a barrier: 0  Comment: [NAME]: yes a barrier. Difficult to keep up. No salary. Respect from peers and leadership. Things in Nigeria upside down. Traffic. Environment. Put in our best concerning the guidelines. [NAME]: agree with [NAME]. Respect from peers and leadership. Learning an uncommon procedure. gaining knowledge is an incentive. but past couple of months. have to come from far places. cost of transport and food have sky rocketed. knowledge and respect are good. [NAME]: inclining towards [NAME] and [NAME]. incentives can come in form as training. have respect from peers. but major barrier if replicate to a new facility. to another OSS. they will expect increase of salary with new procedure or incentive. will be a barrier. | 4/4 |
| 21 | Goals and Feedback | Goals are not clearly shared in the TRUST clinic and team members don’t get feedback on progress toward any goals. | This is a barrier: 0  Not a barrier: 4  Comment: unanimous | 4/4 |
| 22 | Learning Climate | Leaders in the TRUST clinic (executives, middle management, supervisors, team leaders) don’t show their weaknesses or ask for help from team members. Team members don’t feel valued or supported to try new things, and don’t feel they have enough time and space to think about and evaluate their practice. | This is a barrier: 0  Not a barrier: 4  Comment: unanimous | 4/4 |
| 23 | Readiness for Implementation | It’s not clear that the TRUST clinic is ready and committed to implement the IANS guidelines. | This is a barrier: 0  Not a barrier: 4  Comment: unanimous | 4/4 |
| 24 | Leadership Engagement | Key leaders or managers in the TRUST clinic are not involved in, committed to, or held responsible for the implementation of the IANS guidelines. | This is a barrier: 0  Not a barrier: 4  Comment: unanimous | 4/4 |
| 25 | Available Resources | There aren’t enough resources (money, training, education, space, time) to support the IANS guidelines. | This is a barrier: 4  Not a barrier: 0  Comment: [NAME]: barrier for training. only training was online and 1 week of hands on (4 days). No other form of training. not getting enough training and education. Don't feel as if need anything for doing the procedure. Need hands on training; other forms of training. [NAME]: availability of funds. since research has budget lines for training. not a barrier to him because in the budget. [NAME]: [NAME] and [NAME] said everything. agree with them. | 4/4 |
| 26 | Access to knowledge and information | Team members don’t have enough access to knowledge and information to help them with putting the guidelines into practice. | This is a barrier: 0  Not a barrier: 4  Comment: unanimous | 4/4 |
| CHARACTERISTICS OF INDIVIDUALS | | |  |  |
| 27 | Knowledge & Beliefs about the Intervention | Team members don’t like the IANS guidelines, don’t understand them, and don’t put a lot of importance on implementing them. | This is a barrier: 0  Not a barrier: 4  Comment: unanimous | 4/4 |
| 28 | Self-efficacy | Team members aren’t confident they can do what’s needed to reach implementation goals. | This is a barrier: 0  Not a barrier: 4  Comment: unanimous | 4/4 |
| 29 | Individual Stage of Change | Team members don’t have the skills or aren’t that excited about continuing to use the IANS guidelines. | This is a barrier: 4  Not a barrier: 0  Comment: [NAME]: dicey. Team members have skills. Barrier. Mixed response. Have skills and feelings to implement guidelines. Need to make changes to reach implementation goals. Not a barrier. We have skills. But not at best possible place. practice makes perfect. more we do. excited implementing it. need to do tweaks. [NAME]: practice makes perfect. we are not 100% perfect, but excited to use IANS training. very much good. [NAME]: slight barrier: trust clinic is unique. influence of international mentor. can't recreate this. | 4/4 |
| 30 | Individual Identification with Organization | Team members aren’t satisfied and aren’t really committed to the TRUST clinic. | This is a barrier: 0  Not a barrier: 4  Comment: unanimous | 4/4 |
| PROCESS | | |  |  |
| 31 | Planning | A plan for implementing the IANS guidelines, including all the steps needed, hasn’t been made or is poorly outlined. | This is a barrier: 0  Not a barrier: 4  Comment: unanimous | 4/4 |
| 32 | Opinion Leaders | Gate-keepers or opinion leaders who can influence other peoples’ attitudes or beliefs toward implementing the IANS guidelines are not involved or supportive. | This is a barrier: 0  Not a barrier: 4  Comment: unanimous | 4/4 |
| 33 | Formally appointed internal implementation leaders | There is no one person(s) responsible for making sure the IANS guidelines are being implemented. | This is a barrier: 0  Not a barrier: 4  Comment: unanimous | 4/4 |
| 34 | Champions | Individuals who act as champions for the IANS guidelines by supporting, marketing, driving, or overcoming any indifference or resistance from key team members, are not involved or supportive. | This is a barrier: 0  Not a barrier: 4  Comment: unanimous | 4/4 |
| 35 | External Change Agents | Individuals from an outside organization who help with decision-making and moving things forward, are not involved or supportive. | This is a barrier: 2  Not a barrier: 2  Comment: [NAME]: barrier, 4 factors of guidelines depend on the online course. need acceptability from external agents (govt, naca, fmoh) need advocacy. Barrier if go beyond trust clinic. How put in resources both material, human and money to make it sustainable. [NAME]: barrier, way it is that implementing in country where population, need govt of Nigeria, fmoh. criteria we use to bring people in does not involve external agents; put persons at risk. [NAME]: not a barrier. [NAME]: not a barrier. | 2/4 |
| 36 | Key Stakeholders | Ways of attracting and involving **key stakeholders** in implementing the IANS guidelines (e.g. posters, pamphlets, information sessions, training, role modelling) have not been developed or don’t work. | This is a barrier: 3  Not a barrier: 1  Comment: [NAME]: not a barrier. [NAME]: barrier. Not involving key stakeholders. Training role modeling. We don't have posters or pamphlets. Not developed or deployed. [NAME]: barrier. Ends beyond clinic. Policy makers. [NAME]: agree barrier. Involvement of stakeholders is limited. | 3/4 |
| 37 | Patients/Customers | Ways of attracting and involving **clients** in implementing the IANS guidelines (e.g. posters, pamphlets, information sessions, training, role modelling) have not been developed or don’t work. | This is a barrier: 0  Not a barrier: 4  Comment: unanimous | 4/4 |
| 38 | Executing | The IANS guidelines have not been implemented as planned. | This is a barrier: 0  Not a barrier: 4  Comment: unanimous | 4/4 |
| 39 | Reflecting & Evaluating | There is little or no feedback about how implementation of the IANS guidelines is going, and no regular meetings to talk about progress or peoples’ experiences are being held. | This is a barrier: 0  Not a barrier: 4  Comment: unanimous | 4/4 |

**IMPACT CFIR Card Game for External Stakeholders**

| Aug.24, 2023.  Time started: 11:50 AM (WAT)  Introduction, presentation and consenting.  Time started CFIR game: 12:22 PM (WAT)  Time ended CFIR game: 1:48 PM (WAT) Indicates an identified barrier | | | | |
| --- | --- | --- | --- | --- |
| INNOVATION CHARACTERISTICS | |  | External Stakeholders (operationalization) | External Stakeholders (data) |
| 1 | Innovation Source | Team members don’t like the IANS guidelines because of who developed them or where they were developed. | This is a barrier: 1  Not a barrier: 7  Did not vote (skip): 0  Comment: may be weary of accepting guidelines done by developed countries. | 1/8 |
| 2 | Evidence Strength & Quality | Team members don’t like the IANS guidelines because they question the research data that says they are helpful for patients. | This is a barrier: 1  Not a barrier: 7  Did not vote (skip): 0  Comment: Because the ANCHOR study was done primarily in the Western countries. | 1/8 |
| 3 | Relative advantage | Team members do not see why the IANS guidelines is better than what they are already doing or other guidelines they could try. | This is a barrier: 0  Not a barrier: 8  Did not vote (skip): 0  Comment: unanimous | 0/8 |
| 4 | Adaptability | Team members don’t think the IANS guidelines can be adapted to local needs. | This is a barrier: 6  Not a barrier: 2  Did not vote (skip): 0  Comment: costs for infrastructures, and training may be a challenge for generalization and adaptation in the general setting. | 6/8 |
| 5 | Trialability | Team members don’t think they can pilot the IANS guidelines or revert to the status quo. | This is a barrier: 0  Not a barrier: 8  Did not vote (skip): 0  Comment: unanimous | 0/8 |
| 6 | Complexity | Team members believe the IANS guidelines are complex because they involve lots of steps, take a long time, and/ or are disruptive. | This is a barrier: 1  Not a barrier: 7  Did not vote (skip): 0  Comment: complex steps may be a potential barrier, but it still has some advantage. | 1/8 |
| 7 | Design Quality and Packaging | Team members believe the IANS guidelines aren’t very good because of the way they have been packaged and presented. | This is a barrier: 7  Not a barrier: 1  Did not vote (skip): 0  Comment: The package and design is a barrier because the training/experts are not readily available in the general health setting. | 7/8 |
| 8 | Cost | Team members think the IANS guidelines would be too expensive to implement. | This is a barrier: 7  Not a barrier: 1  Did not vote (skip): 0  Comment: training, travelling, instruments to set up are expensive without external sponsors. | 7/8 |
| OUTER SETTING | |  |  |  |
| 9 | Patient Needs & Resources | The IANS guidelines don’t account for what patients in Nigeria need, or don’t address how to meet those needs. | This is a barrier: 8  Not a barrier: 0  Did not vote (skip): 0  Comment: a strong barrier because the guidelines do not address the multiple needs of patients to prioritize what is most needed and how best to address them. | 8/8 |
| 10 | Cosmopolitanism | The IANS organization is not well connected to partners in Nigeria. | This is a barrier: 8  Not a barrier: 0  Did not vote (skip): 0  Comment: The IANS organization is limited to specific organization and partners in Nigeria. | 8/8 |
| 11 | Peer Pressure | There is no pressure to implement the IANS guidelines because other clinics in Nigeria are not using them. | This is a barrier: 8  Not a barrier: 0  Did not vote (skip): 0  Comment: This is a barrier because it is new, and there are few clinics to emulate. | 8/8 |
| 12 | External Policy & Incentives | There are no clinic or outside rules that say the IANS guidelines must be adopted. Or the rules in place get in the way of adopting the guidelines. | This is a barrier: 8  Not a barrier: 0  Did not vote (skip): 0  Comment: a strong policy enforcing it will make it acceptable. | 8/8 |
| INNER SETTING | |  |  |  |
| 13 | Structural Characteristics | The TRUST clinic size, history, or leadership/staffing structure get in the way of adopting the IANS guidelines. | Participants skipped due to inadequate knowledge. | N/A |
| 14 | Networks & Communications | It's hard to implement the IANS guidelines because of poor communication in the TRUST clinic. | Participants skipped due to inadequate knowledge. | N/A |
| 15 | Culture | The culture of the TRUST clinic (the ways things are done, the values, and the way of thinking) makes implementing the IANS guidelines more difficult. | Participants skipped due to inadequate knowledge. | N/A |
| 16 | Implementation Climate | It's hard to change things in the TRUST clinic because people are either not willing to make changes or they do not see the benefit of trying something new. | Participants skipped due to inadequate knowledge. | N/A |
| 17 | Tension for Change | Team members do not think the IANS guidelines are needed. | Participants skipped due to inadequate knowledge. | N/A |
| 18 | Compatibility | There isn’t a good fit between the IANS guidelines and the existing workflows, systems, norms and values of the TRUST clinic. | Participants skipped due to inadequate knowledge. | N/A |
| 19 | Relative Priority | Team members feel that implementing the IANS guidelines isn’t given much importance because other activities have a higher priority. | Participants skipped due to inadequate knowledge. | N/A |
| 20 | Organizational Incentives & Rewards | There is no incentive or reward if adopt the IANS guidelines, such as salary raises or respect from peers or leadership. | Participants skipped due to inadequate knowledge. | N/A |
| 21 | Goals and Feedback | Goals are not clearly shared in the TRUST clinic and team members don’t get feedback on progress toward any goals. | Participants skipped due to inadequate knowledge. | N/A |
| 22 | Learning Climate | Leaders in the TRUST clinic (executives, middle management, supervisors, team leaders) don’t show their weaknesses or ask for help from team members. Team members don’t feel valued or supported to try new things, and don’t feel they have enough time and space to think about and evaluate their practice. | Participants skipped due to inadequate knowledge. | N/A |
| 23 | Readiness for Implementation | It’s not clear that the TRUST clinic is ready and committed to implement the IANS guidelines. | Participants skipped due to inadequate knowledge. | N/A |
| 24 | Leadership Engagement | Key leaders or managers in the TRUST clinic are not involved in, committed to, or held responsible for the implementation of the IANS guidelines. | Participants skipped due to inadequate knowledge. | N/A |
| 25 | Available Resources | There aren’t enough resources (money, training, education, space, time) to support the IANS guidelines. | Participants skipped due to inadequate knowledge. | N/A |
| 26 | Access to knowledge and information | Team members don’t have enough access to knowledge and information to help them with putting the guidelines into practice. | Participants skipped due to inadequate knowledge. | N/A |
| CHARACTERISTICS OF INDIVIDUALS | | |  |  |
| 27 | Knowledge & Beliefs about the Intervention | Team members don’t like the IANS guidelines, don’t understand them, and don’t put a lot of importance on implementing them. | This is a barrier: 0  Not a barrier: 8  Did not vote (skip): 0  Comment: unanimous | 0/8 |
| 28 | Self-efficacy | Team members aren’t confident they can do what’s needed to reach implementation goals. | This is a barrier: 0  Not a barrier: 8  Did not vote (skip): 0  Comment: unanimous | 0/8 |
| 29 | Individual Stage of Change | Team members don’t have the skills or aren’t that excited about continuing to use the IANS guidelines. | This is a barrier: 0  Not a barrier: 8  Did not vote (skip): 0  Comment: unanimous | 0/8 |
| 30 | Individual Identification with Organization | Team members aren’t satisfied and aren’t really committed to the TRUST clinic. | This is a barrier: 0  Not a barrier: 8  Did not vote (skip): 0  Comment: unanimous | 0/8 |
| PROCESS | | |  |  |
| 31 | Planning | A plan for implementing the IANS guidelines, including all the steps needed, hasn’t been made or is poorly outlined. | This is a barrier: 0  Not a barrier: 8  Did not vote (skip): 0  Comment: unanimous | 0/8 |
| 32 | Opinion Leaders | Gate-keepers or opinion leaders who can influence other peoples’ attitudes or beliefs toward implementing the IANS guidelines are not involved or supportive. | This is a barrier: 0  Not a barrier: 8  Did not vote (skip): 0  Comment: unanimous | 0/8 |
| 33 | Formally appointed internal implementation leaders | There is no one person(s) responsible for making sure the IANS guidelines are being implemented. | This is a barrier: 0  Not a barrier: 8  Did not vote (skip): 0  Comment: unanimous | 0/8 |
| 34 | Champions | Individuals who act as champions for the IANS guidelines by supporting, marketing, driving, or overcoming any indifference or resistance from key team members, are not involved or supportive. | This is a barrier: 0  Not a barrier: 8  Did not vote (skip): 0  Comment: unanimous | 0/8 |
| 35 | External Change Agents | Individuals from an outside organization who help with decision-making and moving things forward, are not involved or supportive. | This is a barrier: 0  Not a barrier: 8  Did not vote (skip): 0  Comment: unanimous | 0/8 |
| 36 | Key Stakeholders | Ways of attracting and involving **key stakeholders** in implementing the IANS guidelines (e.g. posters, pamphlets, information sessions, training, role modelling) have not been developed or don’t work. | This is a barrier: 0  Not a barrier: 8  Did not vote (skip): 0  Comment: unanimous | 0/8 |
| 37 | Patients/Customers | Ways of attracting and involving **clients** in implementing the IANS guidelines (e.g. posters, pamphlets, information sessions, training, role modelling) have not been developed or don’t work. | This is a barrier: 0  Not a barrier: 8  Did not vote (skip): 0  Comment: unanimous | 0/8 |
| 38 | Executing | The IANS guidelines have not been implemented as planned. | This is a barrier: 0  Not a barrier: 8  Did not vote (skip): 0  Comment: unanimous | 0/8 |
| 39 | Reflecting & Evaluating | There is little or no feedback about how implementation of the IANS guidelines is going, and no regular meetings to talk about progress or peoples’ experiences are being held. | This is a barrier: 0  Not a barrier: 8  Did not vote (skip): 0  Comment: unanimous | 0/8 |

**IMPACT CFIR Card Game for Screened Patients**

| Aug.22, 2023.  Time started: 11:00 AM (WAT)  Introduction, presentation and consenting.  Time started CFIR game: 11:43 AM (WAT)  Time ended CFIR game: 2:04 PM (WAT)  Indicates an identified barrier | | | | |
| --- | --- | --- | --- | --- |
| INNOVATION CHARACTERISTICS | |  | Participants (operationalization) | Participants (data) |
| 1 | Innovation Source | Team members don’t like the IANS guidelines because of who developed them or where they were developed. | This is a barrier: 2  Not a barrier: 6  Comment: this procedure is unique and may not be easy to convince people here in Nigeria, and because it is not developed in the African way. | 2/8 |
| 2 | Evidence Strength & Quality | Team members don’t like the IANS guidelines because they question the research data that says they are helpful for patients. | This is a barrier: 0  Not a barrier: 8  Comment: unanimous | 0/8 |
| 3 | Relative advantage | Team members do not see why the IANS guidelines is better than what they are already doing or other guidelines they could try. | Participants skipped due to inadequate knowledge. | N/A |
| 4 | Adaptability | Team members don’t think the IANS guidelines can be adapted to local needs. | This is a barrier: 5  Not a barrier: 3  Comment: other places may not be a safe space enough to adapt it, and proper training of the staff may be an issue | 5/8 |
| 5 | Trialability | Team members don’t think they can pilot the IANS guidelines or revert to the status quo. | Participants skipped due to inadequate knowledge. | N/A |
| 6 | Complexity | Team members believe the IANS guidelines are complex because they involve lots of steps, take a long time, and/ or are disruptive. | Participants skipped due to inadequate knowledge. | N/A |
| 7 | Design Quality and Packaging | Team members believe the IANS guidelines aren’t very good because of the way they have been packaged and presented. | Participants skipped due to inadequate knowledge. | N/A |
| 8 | Cost | Team members think the IANS guidelines would be too expensive to implement. | Participants skipped due to inadequate knowledge. | N/A |
| OUTER SETTING | |  |  |  |
| 9 | Patient Needs & Resources | The IANS guidelines don’t account for what patients in Nigeria need, or don’t address how to meet those needs. | This is a barrier: 0  Not a barrier: 8  Comment: unanimous | 0/8 |
| 10 | Cosmopolitanism | The IANS organization is not well connected to partners in Nigeria. | This is a barrier: N/A  Not a barrier: 1  Comment: 7 participants do not have idea of connections to partners | 0/1 |
| 11 | Peer Pressure | There is no pressure to implement the IANS guidelines because other clinics in Nigeria are not using them. | This is a barrier: 8  Not a barrier: 0  Comment: we are resistant to change without pressure | 8/8 |
| 12 | External Policy & Incentives | There are no clinic or outside rules that say the IANS guidelines must be adopted. Or the rules in place get in the way of adopting the guidelines. | This is a barrier: 8  Not a barrier: 0  Comment: unanimous | 8/8 |
| INNER SETTING | |  |  |  |
| 13 | Structural Characteristics | The TRUST clinic size, history, or leadership/staffing structure get in the way of adopting the IANS guidelines. | This is a barrier: 1  Not a barrier: 7  Comment: trusting of the staff may be a barrier even with a trained staff | 1/8 |
| 14 | Networks & Communications | It's hard to implement the IANS guidelines because of poor communication in the TRUST clinic. | This is a barrier: N/A  Not a barrier: 1  Comment: 7 participants do not have idea the extent of communication in the clinic | 0/1 |
| 15 | Culture | The culture of the TRUST clinic (the ways things are done, the values, and the way of thinking) makes implementing the IANS guidelines more difficult. | This is a barrier: 0  Not a barrier: 8  Comment: unanimous | 0/8 |
| 16 | Implementation Climate | It's hard to change things in the TRUST clinic because people are either not willing to make changes or they do not see the benefit of trying something new. | Participants skipped due to inadequate knowledge. | N/A |
| 17 | Tension for Change | Team members do not think the IANS guidelines are needed. | This is a barrier: 0  Not a barrier: 8  Comment: unanimous | 0/8 |
| 18 | Compatibility | There isn’t a good fit between the IANS guidelines and the existing workflows, systems, norms and values of the TRUST clinic. | This is a barrier: 0  Not a barrier: 8  Comment: unanimous | 0/8 |
| 19 | Relative Priority | Team members feel that implementing the IANS guidelines isn’t given much importance because other activities have a higher priority. | This is a barrier: 0  Not a barrier: 8  Comment: unanimous | 0/8 |
| 20 | Organizational Incentives & Rewards | There is no incentive or reward if adopt the IANS guidelines, such as salary raises or respect from peers or leadership. | Participants skipped due to inadequate knowledge. | N/A |
| 21 | Goals and Feedback | Goals are not clearly shared in the TRUST clinic and team members don’t get feedback on progress toward any goals. | Participants skipped due to inadequate knowledge. | N/A |
| 22 | Learning Climate | Leaders in the TRUST clinic (executives, middle management, supervisors, team leaders) don’t show their weaknesses or ask for help from team members. Team members don’t feel valued or supported to try new things, and don’t feel they have enough time and space to think about and evaluate their practice. | Participants skipped due to inadequate knowledge. | N/A |
| 23 | Readiness for Implementation | It’s not clear that the TRUST clinic is ready and committed to implement the IANS guidelines. | This is a barrier: 0  Not a barrier: 8  Comment: unanimous | 0/8 |
| 24 | Leadership Engagement | Key leaders or managers in the TRUST clinic are not involved in, committed to, or held responsible for the implementation of the IANS guidelines. | Participants skipped due to inadequate knowledge. | N/A |
| 25 | Available Resources | There aren’t enough resources (money, training, education, space, time) to support the IANS guidelines. | Participants skipped due to inadequate knowledge. | N/A |
| 26 | Access to knowledge and information | Team members don’t have enough access to knowledge and information to help them with putting the guidelines into practice. | Participants skipped due to inadequate knowledge. | N/A |
| CHARACTERISTICS OF INDIVIDUALS | | |  |  |
| 27 | Knowledge & Beliefs about the Intervention | Team members don’t like the IANS guidelines, don’t understand them, and don’t put a lot of importance on implementing them. | This is a barrier: 0  Not a barrier: 8  Comment: unanimous | 0/8 |
| 28 | Self-efficacy | Team members aren’t confident they can do what’s needed to reach implementation goals. | This is a barrier: 0  Not a barrier: 8  Comment: unanimous | 0/8 |
| 29 | Individual Stage of Change | Team members don’t have the skills or aren’t that excited about continuing to use the IANS guidelines. | This is a barrier: 0  Not a barrier: 8  Comment: unanimous | 0/8 |
| 30 | Individual Identification with Organization | Team members aren’t satisfied and aren’t really committed to the TRUST clinic. | This is a barrier: 0  Not a barrier: 8  Comment: unanimous | 0/8 |
| PROCESS | | |  |  |
| 31 | Planning | A plan for implementing the IANS guidelines, including all the steps needed, hasn’t been made or is poorly outlined. | Participants skipped due to inadequate knowledge. | N/A |
| 32 | Opinion Leaders | Gate-keepers or opinion leaders who can influence other peoples’ attitudes or beliefs toward implementing the IANS guidelines are not involved or supportive. | Participants skipped due to inadequate knowledge. | N/A |
| 33 | Formally appointed internal implementation leaders | There is no one person(s) responsible for making sure the IANS guidelines are being implemented. | This is a barrier: N/A  Not a barrier: 1  Comment: 7 participants do not have idea if anyone is responsible to implement it. | 0/1 |
| 34 | Champions | Individuals who act as champions for the IANS guidelines by supporting, marketing, driving, or overcoming any indifference or resistance from key team members, are not involved or supportive. | Participants skipped due to inadequate knowledge. | N/A |
| 35 | External Change Agents | Individuals from an outside organization who help with decision-making and moving things forward, are not involved or supportive. | Participants skipped due to inadequate knowledge. | N/A |
| 36 | Key Stakeholders | Ways of attracting and involving **key stakeholders** in implementing the IANS guidelines (e.g. posters, pamphlets, information sessions, training, role modelling) have not been developed or don’t work. | Participants skipped due to inadequate knowledge. | N/A |
| 37 | Patients/Customers | Ways of attracting and involving **clients** in implementing the IANS guidelines (e.g. posters, pamphlets, information sessions, training, role modelling) have not been developed or don’t work. | This is a barrier: 0  Not a barrier: 8  Comment: unanimous | 0/8 |
| 38 | Executing | The IANS guidelines have not been implemented as planned. | Participants skipped due to inadequate knowledge. | N/A |
| 39 | Reflecting & Evaluating | There is little or no feedback about how implementation of the IANS guidelines is going, and no regular meetings to talk about progress or peoples’ experiences are being held. | Participants skipped due to inadequate knowledge. | N/A |
